# A Process-Based Approach to Transtheoretical Clinical Research and Training

**DOI:** 10.32872/cpe.11987

**Published:** 2024-04-26

**Authors:** Stefan G. Hofmann, Steven C. Hayes

**Affiliations:** 1 Department of Psychology, Philipps-University of Marburg, Marburg, Germany; 2 Department of Psychology, University of Nevada, Reno, NV, USA; Philipps-University of Marburg, Marburg, Germany

**Keywords:** psychopathology, psychotherapy, evolutionary science, adaptation, context, processes, dynamic networks

## Abstract

**Background:**

The science and practice of psychopathology and psychological intervention of today is more like an island archipelago than it is a single land mass, and connections between different traditions are both limited and fraught with misunderstanding.

**Method:**

Our analysis and solution to the problem is process-based therapy (PBT). PBT defines psychopathology as failed adaptation processes to a given context. Therapy involves adaptation through context-dependent or context-altering applications of biopsychosocial strategies that allows a goal to be met.

**Results:**

This coherent approach to more transtheoretical and integrative concepts of clinical training and practice provides a firm foundation by targeting biopsychosocial processes of change, analyzing these processes using an idiographic complex network analytic approach, and organizing findings on the intellectual agora of multi-dimensional and multi-level evolutionary science.

**Conclusion:**

PBT is a new empirical form of functional analysis, resulting in interventions and trainings that are built on elements or kernels of direct relevance to client’s specific needs. In PBT, case formulation continues as long as treatment persists.

## Highlights

- Clinical psychology and psychiatry are too syndrome focused.
- This limits the potential of the interventions.
- An idiographic, context-sensitive approach will resolve this impasse.
- Process-based therapy offers such an approach.

Most clinical concepts in wide use in psychotherapy have emerged from particular applied theoretical positions or technological approaches, linked to specific normative measures, and clusters of treatment methods. It is not unusual for these to be especially focused on particular disorders, populations, or treatment settings. Methods differ in the emphasis across dimensions of human experience: this method is more cognitive, that is more bodily focused, while another emphasizes social relationships. Training occurs by experts in these clusters, and entire training programs in major universities are often readily characterized in that way.

In effect, the science and practice of psychopathology and psychological intervention is more like an island archipelago than it is a single land mass, and connections between different traditions are both limited and fraught with misunderstanding. There has long been pressure and regular efforts to build a great sense of cooperation across these distinct theoretical and practical clusters, especially by practicing clinicians, but the barriers to doing so are considerable. Clinical researchers and treatment developers may give lip service to the importance of transtheoretical conversations, but the methods they develop and test are often distinctive as compared to others.

Over the last decade, however, a new focus and analytic approach has emerged that now has a growing record of fostering evidence-based transtheoretical and integrative concepts in psychotherapy research, and in clinical training and practice. This focus breaks down barriers between the various “schools” of psychological therapy, and provides a new and more functional approach to psychopathology and intervention concepts and methods. It promises to profoundly transform the future of scientifically based clinical training and practice.

We named this approach *Process-based Therapy* (PBT: Hayes & Hofmann, 2021; Hayes, Hofmann, & Ciarrochi, 2020; Hofmann & Hayes, 2019; Hofmann, Hayes, & Lorscheid, 2021). PCT is not a new therapy as such – it is a new vision of the central tasks that need to be accomplished by evidence-based intervention science. PBT has a characteristic target, analytic approach, and meta-model. Its target is the understanding of biopsychosocial processes of change and how they can be modified by treatment components or kernels to help accomplish the goals of the client. Its analytic approach is to measure, predict, and influence idiographically assessed processes of change with high levels of precision, scope, depth and to generate nomothetic generalizations only to the degree to which doing so increases idiographic fit within complex networks. Its meta-model is meant to create a kind of intellectual agora based on a common language of multi-dimensional and multi-level evolutionary science.

PBT defines psychopathology as failed adaptation processes to a given context. PBT applies socially extended principles of contextual adaptation from evolutionary science to psychopathology and psychological interventions, hereby focusing on the human ability to adapt to or alter environmental challenges through variation (in contrast to psychology inflexibility), selection of adequate strategies, and retention of successful strategies at the psychological, biophysiological, sociocultural levels of analysis (Hayes, Hofmann, & Ciarrochi, 2020; Hayes, Hofmann, & Wilson, 2020). Broadly defined, adaptation is the context-dependent or context-altering application of biopsychosocial strategies that allows a goal to be met, whereas psychopathology is the maladaptation of these processes. Maladaptation can include a larger failure to create a more adaptive context itself, so the focus on adaptation is not passive and it is not socially blaming or irresponsible. Entire families or communities can in principle be pathological.

In each level of organizational complexity, specific dimensions can be involved in healthy variation, that is selected and retained in context. For example, the biophysiological level might involve genes, epigenes, brain circuits, or organ systems, among various others. A psychological level might include dimensions of affect, cognition, self, attention, motivation, and overt behavior, among other dimensions. There are no hard and fast divisions among dimensions – the point is that the extended revolutionary meta-model can accommodate a variety of useful dimensions and levels of processes of change.

**Figure 1:**
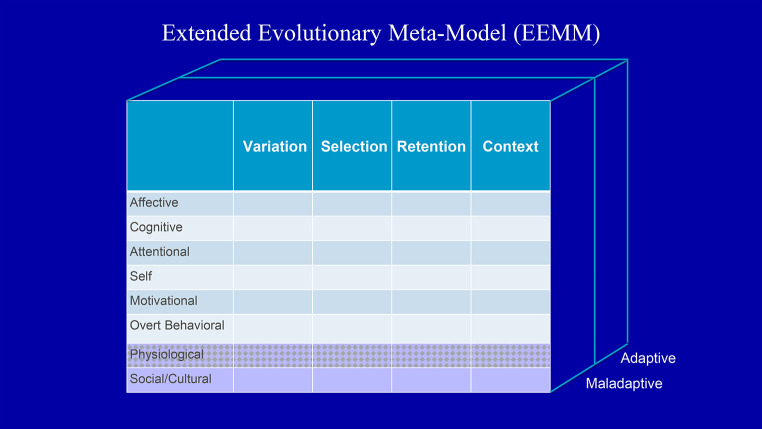
A Conceptual Space for the Examination of Adaptive and Maladaptive Change Processes Provided by Modern Multi-Dimensional, Multi-Level Evolution Science *Note.* The bottom two rows represent nested levels of analysis relevant to the psychological level – although not the topic of this paper a full explication of these levels would require similar matrices of their own. Copyright Steven C. Hayes and Stefan G. Hofmann.

This model has been termed an Extended Evolutionary Meta-Model (EEMM; Hayes et al., 2019; see Figure 1), because it can serve as a meta-model for more specific, independent psychotherapeutic schools, and because of its roots in the knowledge on evolutionary science (Badcock, 2012) applied to the individual in all of its socially-situated complexity (Hofmann, Curtiss, & Hayes, 2020; Hofmann, Curtiss, & McNally, 2016; see Ong et al., 2022 for a recent case description).

It is important to understand that a meta-model is not a new model. It is a model of models. Consider any row of the EEMM – say, the cognitive dimension – to begin to explore why a meta-model is helpful to a transtheoretical appoach.

A given model of psychopathology or treatment may emphasize that persons who develop psychological problems tend to have characteristic cognitive styles or modes of thinking. Perhaps these styles are rigid, irrational, or over-generalized, for example. These are theoretically grounded ways of speaking about how given cognitions or cognitive styles can be insensitive to the actual context or fail to vary when a wider range of possibilities need to be considered. Understanding the person's history might lead to an understanding about why these styles became dominant for a particular person, that is, how they were selected and retained. For example, a person may have had critical parents who modelled irrational thinking styles or who might have reverted to over-generalizations to avoid criticisms by others.

The EEMM is not meant as a substitute for a specific model – it is instead meant as a generic way of considering them and studying them in a transtheoretical fashion. If another specific model emphasizes that cognitive rigidity comes from seeing thoughts as facts, it would be an easy matter to study this idea in comparison to cognitive over-generalization in a given instance. Two theorists might feel compelled to compete if an entire model is at stake, but when specific processes are being compared, it is far easier to have shared interests in any given outcome.

## Idionomic Versus Normative “Latent Disease” Analysis

Analytically, a PBT approach is a radical departure from the traditional latent disease model of psychiatry. It relies on network analysis as a form of functional analysis using the framework of evolutionary science.

In a latent disease model, the signs and symptoms of psychopathology in a given syndrome for a given individual are meant to orient towards underlying entities that are assumed to be driving the particular features that are seen. Latent diseases are inherently normative and categorical. The statistical methods used in traditional psychometric evaluations of measures rely heavily on consistencies between people as the metric for evaluating consistencies across persons that are assumed to be driven by underlying variables that cannot be directly measured but must be inferred. Clinically speaking, after assessment people are grouped in diagnostic de-individualized categories (Greenhalgh et al., 2014). In line with this, particular collections of data, theories, and interventions are utilized with the intention of encompassing and benefiting the entire group. Such labels are commonly found in traditional CBT protocols. The latent disease model gives priority to the prescribed symptoms and syndromes rather the psychological processes underlying psychopathology and mental health. This view tends to reduce human suffering to brain abnormalities and biological dysfunctions and de-emphasize the importance of the biopsychosocial context of the individual (Greenhalgh et al., 2014). Despite the increased transdiagnostic focus of CBT approaches as process-based approach gains strength (Hayes & Hofmann, 2021), narrow attention to the patient’s specific symptoms and normative views of presenting problems remains a main feature of CBT case formulation and treatment delivery.

It is increasingly apparent that the central tendencies of groups do a very poor job of modeling individual life trajectories. Statistical physics long ago proved the ergodic theorem, which suggests that the measurement of a collection of elements can adequately reflect the behavior of individual elements only if the behavior involved is stationary and all individual elements share the same dynamic model. Statisticians agree that this ergodicity is an underlying assumption of common biostatistical methods but by definition processes of change are not stationary, and they unfold in different ways at different times and different people.

In a PBT approach, this problem is avoided through idiographic analyses that model the relation of processes of change as they bear on particular outcomes within the person over time. Only then are individual results related to those of other people. Relations that become evident by extending the analysis to the nomothetic or group level are retained if and only if they improve ideographic fit for most people. This is what is meant by the neologism “idionomic analysis”, offered as a substitute for “normative analysis.”

## Functional Analysis in an Idionomic Approach

Because processes of change interact, process-based therapists conduct functional analyses through contextual sensitive idionomic network assessment. Psychopathology is represented as an idionomic network of problems, conditions, and processes. The goal of PBT is to help clients replace their maladaptive networks with adaptive networks. This is done by strengthening processes that promote well-being while moving toward desirable goals and values.

Early functional analysis and later CBT case formulations (Persons, 2008; Persons et al., 2013) were important steps toward the translation of general processes of change to individual applications. According to PBT, we ask: What core biopsychosocial processes should be targeted with this client given this goal in this situation, and how can they most efficiently and effectively be changed? (Hofmann & Hayes, 2019, p. 38). PBT does not demarcate case conceptualization, assessment and treatment. PBT visualizes a client’s problem as a dynamic network that is maintained through maladaptive processes. Once we understand them, we can effectively intervene. This network is not static, but dynamic and changes with time and treatment. Therefore, high density data monitoring are essential, such as ecological momentary assessment (EMA), dynamic network analysis, and time-series analysis. Examples of some available methods that can be taken in clinical settings are frequent measures of processes taken in session and between sessions, and measures of social, psychological, and physical context (Hayes et al., 2019).

In that way, frequent, contextually focused assessment sets the stage for the creation of a comprehensive empirical form of functional analysis for each client. That is, idionomic analysis of longitudinal assessments leads to the identification of relevant and controllable functional relations to an individual’s specific behavioral targets (Haynes & O’Brien, 1990).

This is quite different from traditional forms of functional analysis that were common in the early days of cognitive and behavioral therapy. In those times, applying principles to individual patterns of behavior was more an art than a science, making replicable case analysis difficult (Hayes & Follette, 1992). Traditional functional analysis was neglected from psychology literature for decades because of that, in addition to the fact that the range of processes considered was too limited, appropriate statistical analytic methods were under-developed, and consequently it was difficult to show superior outcomes from functional analysis for addressing human suffering. In recent decades, newer forms of CBT have reemphasized a functional approach (Hayes, Hofmann, & Ciarrochi, 2020), and research has expanded our clarity about the key processes of change that need to be targeted (Hayes, Ciarrochi, Hofmann, Chin, & Sahdra, 2022), revitalizing a functional analytic approach. Additionally, interventions based on a functional-analytic assessment have demonstrated utility in improving clinical outcomes of some conditions (Ghaderi, 2006; Hurl et al., 2016). It is particularly worth noting that every significant mediator of a randomized control trial of a psychosocial method focused on a mental health outcome, easily fits within the EEMM (Hayes, Ciarrochi, et al., 2022). This means that the EEMM is in fact the intellectual agora sought by PBT: it provides a stable transtheoretical ground for all current approaches to processes of change.

## From Packages to Kernels in Personalized Interventions

PBT is based on the idea that efficient and effective intervention should be based on the individual’s unique biopsychosocial characteristics, goals, and needs. In other words, the dynamic process-based case formulation can and should lead to specific treatment elements or kernels. PBT rejects the a priori focus on symptoms from DSM or ICD-defined syndromes and instead focuses on processes that ameliorate problems or promote prosperity when positive goals are ascendant. Rather than just reduction in symptoms, the aim of PBT is to strengthen processes that promote well-being in accordance to the clients’ values and goals.

We have a great deal to learn about how well treatment elements or components modify processes of change, but systematic reviews of the ability of treatment kernels to do so are already available in some areas (e.g., Levin et al., 2012). The claim that the EEMM can serve as an agora for a transtheoretical approach is also strengthened by evidence that all current positive psychology methods and models also readily fit within the EEMM (Ciarrochi et al., 2022).

## Concluding Thoughts

Broadly-cast transtheoretical approaches to processes of change should lead to an increased ability to share treatment methods without losing intervention coherence. Said in another way, instead of vapid eclecticism, a PBT approach encourages researchers and practitioners to develop broader models of change, to communicate across theoretical boundaries about overlapping interests in processes of change, and to share intervention kernels that successfully alter idiographically relevant biopsychosocial processes. This approach allows the strengths that come from clarity about philosophical assumptions and basic or applied theory, on the one hand, while reaping the benefits that can come from theoretical consilience and cooperation, on the other.
